# Oral vitamin A supplementation in preterm infants to improve health outcomes: A systematic review and meta-analysis

**DOI:** 10.1371/journal.pone.0265876

**Published:** 2022-04-04

**Authors:** Nanthida Phattraprayoon, Teerapat Ungtrakul, Kamonwan Soonklang, Paweena Susantitaphong

**Affiliations:** 1 Princess Srisavangavadhana College of Medicine, Chulabhorn Royal Academy, Bangkok, Thailand; 2 Centre of Learning and Research in Celebration of HRH Princess Chulabhorn’s 60 Birthday Anniversary, Chulabhorn Royal Academy, Bangkok, Thailand; 3 Division of Nephrology, Department of Medicine, Faculty of Medicine, King Chulalongkorn Memorial Hospital, Chulalongkorn University, Bangkok, Thailand; 4 Research Unit for Metabolic Bone Disease in CKD Patients, Faculty of Medicine, Chulalongkorn University, Bangkok, Thailand; Monroe Carell Junior Children’s Hospital at Vanderbilt, UNITED STATES

## Abstract

**Objective:**

To determine the effects of oral vitamin A supplementation on clinical outcomes in preterm infants.

**Design:**

We conducted the meta-analysis by searching PubMed/Medline, Scopus, Embase, CINAHL, and the Cochrane Library databases from inception to 12 August 2021, including reference lists of retrieved articles. Only randomized controlled trials (RCTs) evaluating the effects of oral vitamin A on premature babies were included. We used a random-effects model to calculate risk ratios (RRs) and weighted mean differences (MDs) with 95% confidence intervals (CIs). We used the GRADE approach to grade evidence quality and assess how oral vitamin A supplementation affects clinical outcomes.

**Main outcomes measures:**

The primary outcomes were respiratory outcomes, including the length of respiratory support, the need for oxygen at 36 weeks postmenstrual age (PMA), and moderate-to-severe bronchopulmonary dysplasia (BPD) at 36 weeks PMA. Secondary outcomes were hospitalization time, vitamin A status, mortality, other related outcomes, and potential adverse drug-related events.

**Results:**

We included four RCTs, with 800 patients total. In all trials, oral vitamin A treatment was compared to a placebo. Oral vitamin A supplementation did not significantly affect mechanical ventilation duration (MD, −1.07 days; 95% CI, −2.98 to 0.83 days), oxygen requirement at 36 weeks PMA (RR, 0.65; 95% CI, 0.33 to 1.31), or moderate-to-severe BPD at 36 weeks PMA (RR, 0.53; 95% CI, 0.07 to 4.17). However, oral vitamin A supplementation yielded a slightly shorter noninvasive ventilation duration (MD, −0.96 days; 95% CI, −1.59 to −0.33 days).

**Conclusions:**

Administering oral vitamin A to preterm newborns did not alter the mechanical ventilation duration, oxygen needed at 36 weeks PMA, moderate-to-severe BPD at 36 weeks PMA, death, or short-term benefits. However, oral vitamin A supplementation may slightly affect the duration of noninvasive respiratory support without adverse drug-related events.

## Introduction

In humans, vitamin A is a vital nutrient that regulates and promotes appropriate immunological function, cellular differentiation and proliferation, respiratory epithelial cell integrity, and formation of photosensitive visual pigments in the retina [1–3]. The mechanism by which vitamin A is transported across the placenta in the third trimester is incomplete in preterm fetuses [3]; thus, preterm birth increases the risk of vitamin A insufficiency [4]. Deprivation of transplacental vitamin A due to delivering at an early gestational age, restricted hepatic storage, inadequate supply, and inadequate postnatal vitamin A intake may contribute to this insufficiency, particularly in infants with extremely low birth weights (ELBWs) and very low birth weights (VLBWs) [4].

A trial that administered 5000 IU of vitamin A intramuscularly (IM) three times weekly for 4 weeks found that this dosage reduced vitamin A deficiency and the risk of chronic lung disease (CLD) in ELBW infants [5]. Darlow et al. [3] pooled all relevant studies [5–15] on IM or oral vitamin A supplementation for a meta-analysis and revealed a slight reduction in the risk of death or oxygen requirements at 1 month of age and the risk of CLD at 36 weeks postmenstrual age (PMA). These authors also found a marginal reduction in the combined results for mortality and CLD. However, administering IM injections to these newborn infants was highly invasive and painful.

Currently, little evidence exists to indicate whether oral vitamin A supplementation may benefit premature infants who are at risk of vitamin A insufficiency [3]. Therefore, we conducted this systematic review and meta-analysis of randomized controlled studies (RCTs) to determine whether oral vitamin A supplementation benefits preterm newborns.

## Methods

### Data sources and searches

We conducted a comprehensive and systematic search of the PubMed/Medline (National Library of Medicine, Bethesda, MD, USA), Scopus, Embase, CINAHL, and Cochrane Library databases from inception to 12 August 2021 to identify eligible articles using the terms “oral or enteral” and “vitamin A” or “vit A” and “supplementation” and “preterm” or “premature” or “neonate” or “infant” or “very low birth weight” or “extremely low birth weight”. Additional research was identified by exploring the reference lists of the resulting publications. This systematic review and meta-analysis were conducted according to the Preferred Reporting Items for Systematic Reviews and Meta-Analyses (PRISMA) [16]. The study protocol was registered with PROSPERO (CRD42021273090).

### Eligibility criteria

Only RCTs and human studies assessing the effectiveness of oral vitamin A supplementation in preterm newborns on clinical outcomes were included in the review, without language restrictions.

### Study selection

Two researchers (N.P. and T.U.) independently screened data from the literature according to the inclusion criteria, study design, methodology, and outcome parameters. Any discrepancies were resolved through discussion with a third researcher (P.S.).

### Data extraction and quality assessment

The following data and results were extracted from the included RCTs: the first author, year of publication, study design, country of origin, participant characteristics, intervention type (including dose, administration route, timing, and duration of intervention), and outcomes such as oxygen demand at 28 days of age and 36 weeks PMA. The study investigators were contacted via email for any missing data, unreported data, or additional details. The quality of the included studies was assessed using the Revised Cochrane Risk-of-Bias assessment for randomized trials [17], which determines whether a study has a low, high, or uncertain risk of bias.

### Data synthesis and statistical analysis

We used a random-effects model to calculate risk ratios (RRs) for categorical variables and weighted mean differences (MDs) for continuous variables to determine the efficacy of oral vitamin A supplementation and associated outcomes. The 95% confidence interval (CI) is presented for each pooled estimate. To determine sources of heterogeneity, we performed a subgroup analysis based on intervention. The statistical heterogeneity of I^2^ at 25%, 50%, and 75%, as determined by the Q-statistic and I^2^ tests, indicated low, moderate, and high heterogeneity among studies, respectively. A funnel plot was used to investigate publication bias if at least ten papers qualified for each outcome. Statistical significance was determined at P<0.05. Review Manager 5 software (RevMan 5, 2014; http://community.cochrane.org/tools/review-production-tools/revman-5) was used to conduct all meta-analyses.

The GRADE technique [18] was used to assess the degrees of certainty of evidence for each outcome, which were classified as high, moderate, low, or very low. The GRADE technique uses five downgrading criteria: risk of bias [19], imprecision [20], inconsistency [21], indirectness [22], and publication bias [23]. The GRADEpro Guideline Development Tool was used to create tables summarizing the findings (http://gradepro.org).

## Results

### Search results

**Fig 1:**
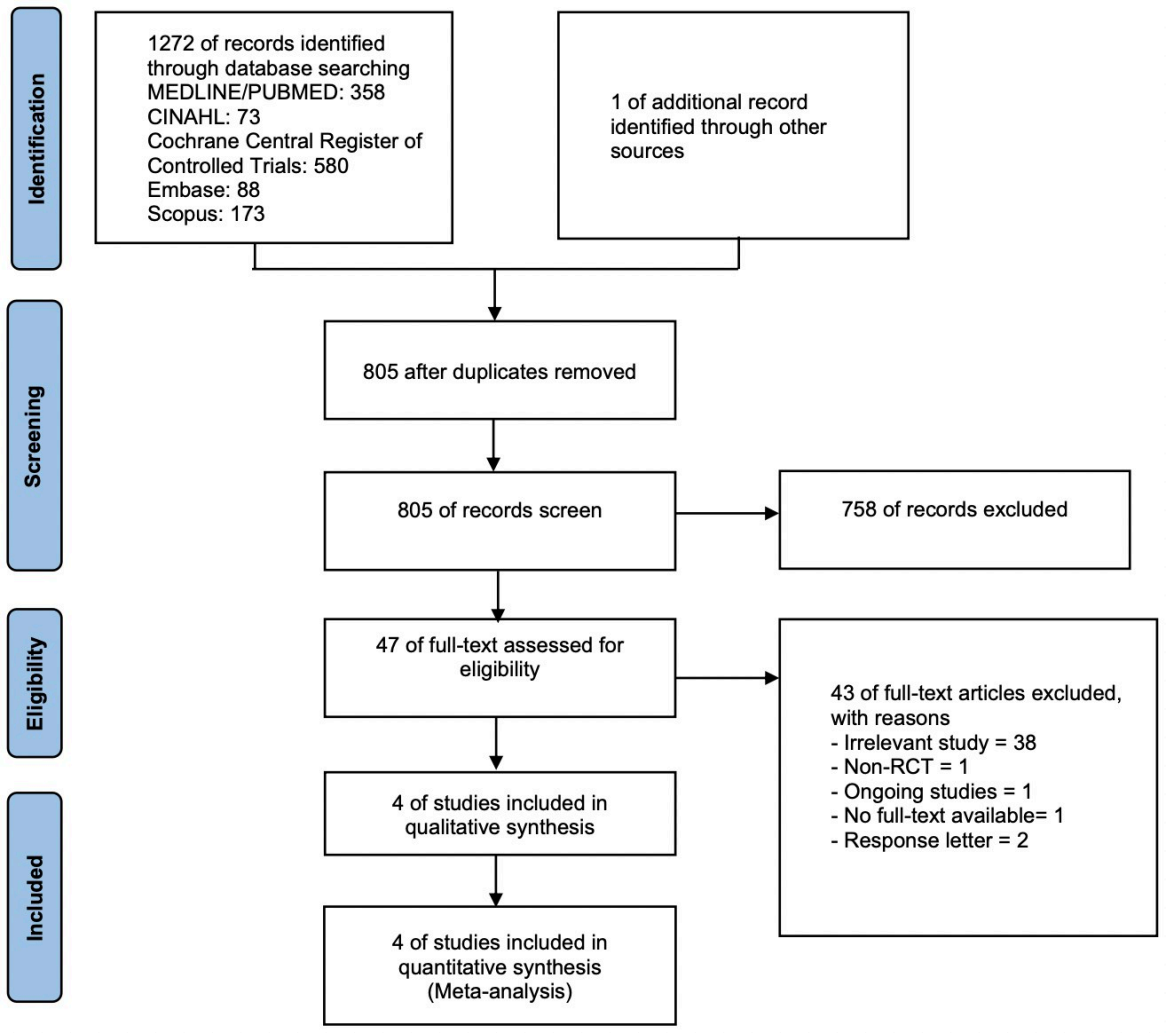
PRISMA flow diagram of the study selection for the systematic review and meta-analysis.

We identified 1272 citations via database searching and one additional record through other sources (Fig 1). After screening the titles and abstracts, we screened 47 full texts. Four studies met our inclusion criteria [15,24–26] and were included in our systematic review and meta-analysis. One additional article [27] was retrieved from the reference lists, but was not of the included studies.

### Study characteristics

We included four RCTs [15, 24–26], with 800 patients total, of whom, 401 received oral vitamin A supplementation, and 399 received a placebo. In each RCT, an oral vitamin A supplement was compared to a placebo. Three RCTs used higher doses of vitamin A: 5000 IU/kg/day [15], 5,000 IU/day [26], and 10,000 IU every other day [24]; one RCT used a lower dose of oral vitamin A at 1500 IU/day [25]. Table 1 summarizes the study characteristics; Supplement 1 in S1 File summarizes the maternal and infant characteristics. One investigation was conducted in India, one in China, one in the United Kingdom, and one in Australia. The included studies were published between 2001 and 2021.

**Table 1:** Characteristics of the included studies.

Study	Wardle et al., 2001 [15]	Basu et al., 2019 [24]	Sun et al., 2020 [25]	Rakshasbhuvankar et al., 2021 [26]
**Study type**	Multicenter RCT	RCT	RCT	RCT
**Location**	United Kingdom	India	China	Australia
**Inclusion criteria**	Preterm < 1000 g Consent before 24 h	VLBW < 1500 g required respiratory support at age before 24 h	Preterm < 28 week, age < 96 h	Preterm < 28 week, age < 72 h
**Exclusion criteria**	Major life threatening congenital anomaly	Major congenital anomaly Life-threatening condition in which immediate oral feeding was contraindicated	Major congenital anomaly Genetic metabolic diseases Congenital TORCH infections Terminal stage of illness	Major congenital anomaly of the respiratory or gastrointestinal tract
**Randomization**	Computer-generated random numbers	Random permuted blocks	Block randomization	Computer-generated randomization table
**Study period**	NR	Jan 2016–Aug 2017	Aug 2015–Dec 2017	Dec 2016–May 2019
**All groups received**	Standard care as protocol	Standard care as protocol	Supportive treatment as protocol of nursery	Standard care as unit protocol
**Control**	Placebo	Placebo	Placebo (soybean oil)	Placebo
**Comparator (1)**	Vitamin A (5000 IU/kg/day) via OGT (postnatal days 1–28)	Oral vitamin A solution (aqueous based, 10,000 IU of retinol/day), alternating days for 28 days, starting at 24 h	Oral vitamin A (1500 IU/day), started with very minimal feeding DOL 4 for 28 days or until discharge	Oral vitamin A (5000 IU/day, started within 24 h until 34 weeks PMA)
**Type of vitamin A supplementation**	NR	Water-soluble	NR	Water-soluble
**VA sources for all infants**	TPN and IL: 23 IU/kg starting on DOL 3 Preterm formula: 1 μg/mL (3.3 IU/mL) BM fortified 0.3 μg/mL (1 IU/mL)—MTV drop: 5000 IU/mL on DOL 14	NR	TPN: 230 IU/kg/day Preterm formula: 1764 IU/day (VA drop 1500 IU/day + formula VA 264 IU/kg/day) Breast milk 2568 IU/day (VA 1500 IU/day + BM 468 IU/kg/day + fortifier VA 600 IU/kg/day)	TPN: 966 IU/kg/day Fortified maternal or donor human milk: 1820 IU/kg/day
**Follow-up** **VA level**	Before VA supplement (baseline) At 12 h, 24 h after VA supplementation Day 7, 28 after VA supplementation	Before VA supplement (baseline) Day 28 after VA supplementation	Before VA supplement (baseline) Day 14, 28 after VA supplementation PMA 36 weeks’ PMA	Day 28 after VA supplementation PMA 34 weeks’ PMA
**Outcomes**	Oxygen requirement at 28 days Oxygen requirement at 36 weeks PMA Mortality Time ventilated(days) Other outcomes IVH with parenchymal involvement NEC required surgery PDA required treatment Sepsis Serum VA levels	Mortality Oxygen requirement at 28 days Safety Tolerability of high dose Serum retinol concentration on recruitment at day 28 Duration of oxygen requirement Duration of respiratory support Other outcomes -Sepsis hs-PDA NEC ≥ stage II IVH ≥ grade II PVL ROP	Mortality Oxygen requirement at 36 weeks PMA (BPD) Serum VA levels Signs of VA toxicity (vomiting, increased intracranial pressure) Other outcomes ROP NEC > stage 2 Sepsis Severe IVH ≥ grade 3 PVL	Oxygen requirement ≥ 28 days and/or required respiratory support at 36 weeks PMA (BPD) Death Duration of oxygen dependence/respiratory support Use of postnatal steroids Proportion of discharged infants with home oxygen Weight gain Other outcomes Sepsis ROP IVH ≥ grade 3 PVL NEC ≥ stage II Adverse effects due to VA (bulging fontanel, hepatomegaly, skin changes)

**Abbreviations**: BPD, bronchopulmonary dysplasia; BM, breast milk; DOL, day of life; h, hour; hs, hemodynamic significant; IL, intralipid; IVH, intraventricular hemorrhage; IU, international unit; MTV, multivitamin; NEC, necrotizing enterocolitis; NR, not reported; OGT, orogastric tube; PDA, patent ductus arteriosus; PMA, postmenstrual age; PVL, periventricular leukomalacia; RCT, randomized controlled trial; ROP, retinopathy of prematurity; TPN, total parenteral nutrition; VA, vitamin A; VLBW, very low birth weight

### Risk-of-bias assessment

Supplement 2 in S1 File summarizes the results of the risk-of-bias assessment of the included RCTs. An allocation sequence was generated in all four RCTs [15, 24–26], and three used concealed allocation [15, 24, 26]. Four studies were double-blind RCTs [15, 24–26]. Loss to follow-up and selective outcome reporting were adequate [15, 24–26].

### Data analyses

**Fig 2:**
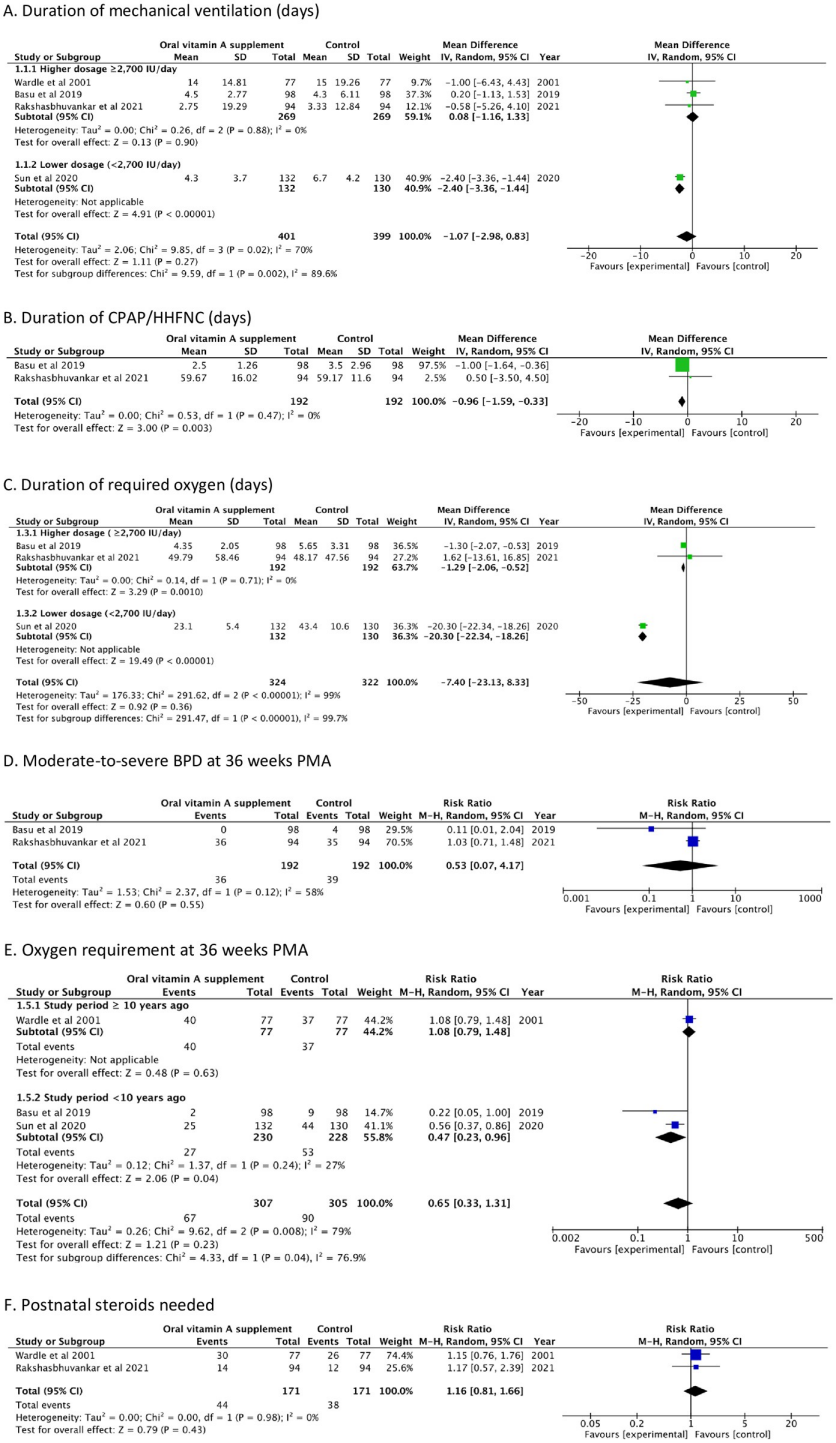
Forest plots of the effects of oral vitamin A supplementation in preterm infants. (A) Duration of mechanical ventilation (days); (B) Duration of CPAP/HHFNC (days); (C) Duration of oxygen requirement (days); (D) Moderate-to-severe BPD at 36 weeks PMA; (E) Oxygen requirement at 36 weeks PMA; (F) Postnatal steroids needed.

**Fig 3:**
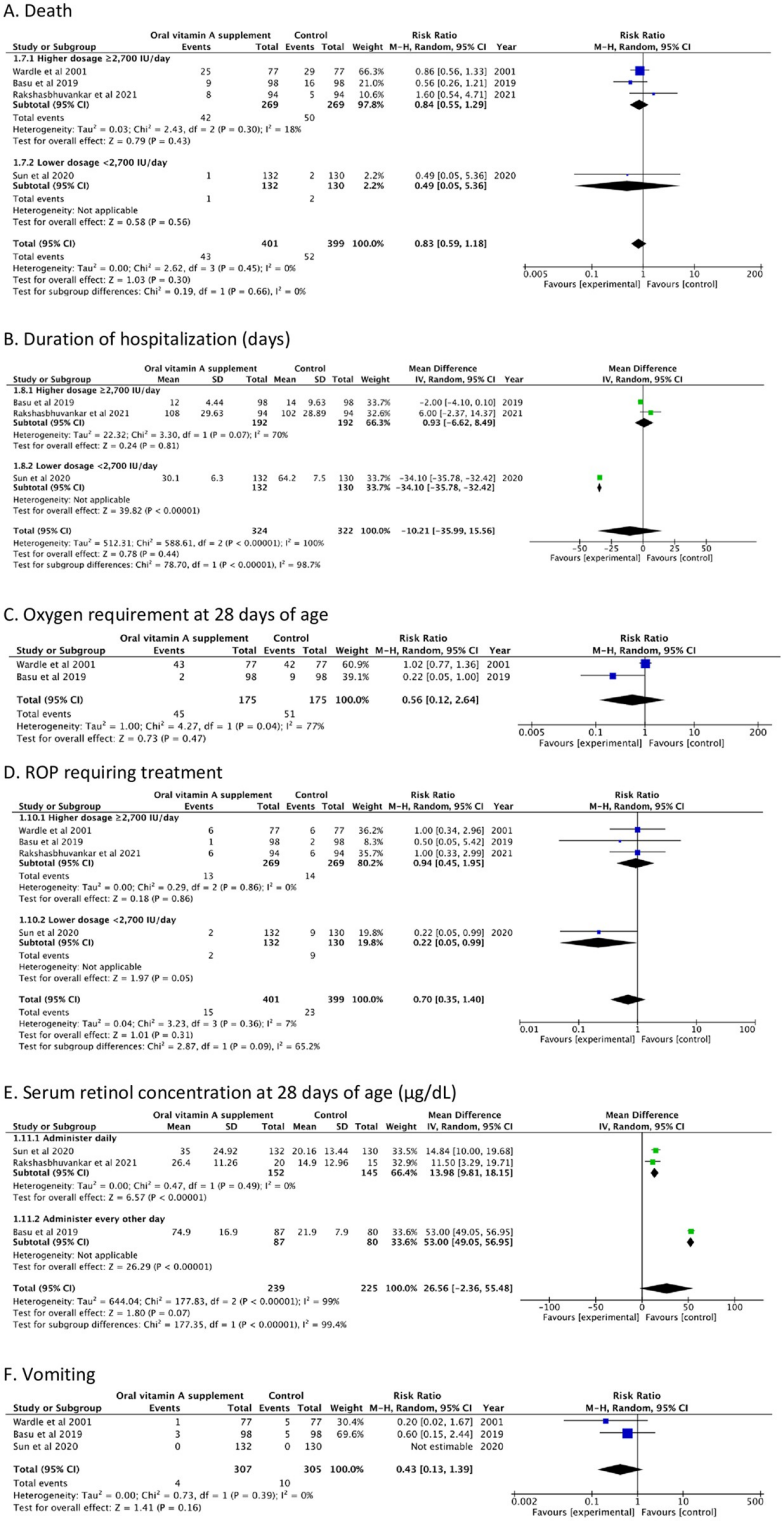
Forest plots of the effects of oral vitamin A supplementation in preterm infants and adverse effects. (A) Death; (B) Duration of hospitalization (days); (C) Oxygen requirement at 28 days of age; (D) ROP requiring treatment; (E) Serum retinol concentration at 28 days of age (μg/dL); (F) Vomiting.

Figs 2 and 3, Table 2, and Supplements 3–5 in S1 File show the effects of oral vitamin A supplementation on clinical outcomes, adverse drug-related events, and serum retinol levels in preterm infants.

**Table 2:** GRADE summary of findings: Effects of oral vitamin A supplementation on clinical outcomes in preterm infants.

Patients or population: Preterm infants Intervention: Oral vitamin A Comparison: Placebo, no treatment, or usual care											
Study design	No. of studies	Certainty assessment					No. of participants		Effect		
		Risk of bias	Inconsistency	Indirectness	Imprecision	Other considerations	Oral vitamin A supplements	Placebo	Estimation of absolute effects		Certainty
									Risk (95% CI)	Absolute (95% CI)	
**Efficacy of oral vitamin A supplementation**											
**Duration of mechanical ventilation (days)**											
RCT	4	Not serious	Serious	Not serious	Serious	None	401	399	-	MD 1.07 lower (2.98 lower to 0.83 higher)	⨁⨁⊝⊝ LOW
**Duration of CPAP or HHFNC (days)**											
RCT	2	Not serious	Not serious	Not serious	Not serious	None	192	192	-	MD 0.96 lower (1.59 lower to 0.33 lower)	⨁⨁⨁⨁ HIGH
**Duration of oxygen requirement (days)**											
RCT	3	Not serious	Serious	Not serious	Serious	None	324	322	-	MD 7.4 lower (23.13 lower to 8.33 higher)	⨁⨁⊝⊝ LOW
**Moderate-to-severe BPD at 36 weeks PMA**											
RCT	2	Not serious	Serious	Not serious	Serious	None	36/192 (18.8%)	39/192 (20.3%)	RR 0.53 (0.07 to 4.17)	95 fewer per 1,000 (from 189 fewer to 644 more)	⨁⨁⊝⊝ LOW
**Oxygen requirement at 36 weeks PMA**											
RCT	3	Not serious	Serious	Not serious	Serious	None	67/307 (21.8%)	90/305 (29.5%)	RR 0.65 (0.33 to 1.31)	103 fewer per 1,000 (from 198 fewer to 91 more)	⨁⨁⊝⊝ LOW
**Postnatal steroids needed**											
RCT	2	Not serious	Not serious	Not serious	Serious	None	44/171 (25.7%)	38/171 (22.2%)	RR 1.16 (0.81 to 1.66)	36 more per 1,000 (from 42 fewer to 147 more)	⨁⨁⨁⊝ MODERATE
**Death**											
RCT	4	Not serious	Not serious	Not serious	Serious	None	43/401 (10.7%)	52/399 (13.0%)	RR 0.83 (0.59 to 1.18)	22 fewer per 1,000 (from 53 fewer to 23 more)	⨁⨁⨁⊝ MODERATE
**Duration of hospitalization (days)**											
RCT	3	Not serious	Serious	Not serious	Serious	None	324	322	-	MD 10.21 lower (35.99 lower to 15.56 higher)	⨁⨁⊝⊝ LOW
**Oxygen requirement at 28 days of age**											
RCT	2	Not serious	Serious	Not serious	Serious	None	45/175 (25.7%)	51/175 (29.1%)	RR 0.56 (0.12 to 2.64)	128 fewer per 1,000 (from 256 fewer to 478 more)	⨁⨁⊝⊝ LOW
**ROP requiring treatment**											
RCT	4	Not serious	Not serious	Not serious	Serious	None	15/401 (3.7%)	23/399 (5.8%)	RR 0.70 (0.35 to 1.40)	17 fewer per 1,000 (from 37 fewer to 23 more)	⨁⨁⨁⊝ MODERATE
**Level of serum retinol concentration**											
**Serum retinol concentration at 28 days old (μg/dL)**											
RCT	3	Not serious	Serious	Not serious	Serious	None	239	225	-	MD 26.56 higher (2.36 lower to 55.48 higher)	⨁⨁⊝⊝ LOW
**Adverse Drug-related Reactions**											
**Vomiting**											
RCT	3	Not serious	Not Serious	Not serious	Serious	None	4/307 (1.3%)	10/305 (3.3%)	RR 0.43 (0.13 to 1.39)	19 fewer per 1,000 (from 29 fewer to 13 more)	⨁⨁⨁⊝ MODERATE

**Abbreviations**: BPD, bronchopulmonary dysplasia; CI, confidence interval; CPAP, continuous positive airway pressure; HHNC, humidified high-flow nasal cannula; MD, mean difference; PMA, postmenstrual age; RCT, randomized controlled trial; ROP, retinopathy of prematurity; RR, risk ratio

#### Duration of mechanical ventilation (days)

Four RCTs provided the duration of mechanical ventilation [15, 24–26]. Oral vitamin A supplementation did not affect the duration of mechanical ventilation, with low-quality evidence (MD, −1.07 days; 95% CI, −2.98 to 0.83 days). Subgroup analysis revealed no effects of higher dosages (Fig 2A, Table 2, and Supplements 3 and 4 in S1 File).

#### Duration of continuous positive airway pressure (CPAP) or humidified high-flow nasal cannula (HHFNC) (days) and duration of oxygen requirement (days)

Two RCTs [24, 26] evaluated the effect of oral vitamin A supplementation on the durations of CPAP or HHFNC use; the durations of using these noninvasive ventilators differed significantly between the oral vitamin A and placebo groups (MD, −0.96 days; 95% CI, −1.59 to −0.33 days) with high-quality evidence.

Three RCTs [24–26] evaluated the duration of required oxygen use; low-quality evidence showed that oral vitamin A supplementation did not affect this duration (MD, −7.4 days; 95% CI, −23.13 to 8.33 days). However, two RCTS found that higher vitamin A doses may reduce the time that preterm infants must remain on oxygen (MD, −1.29 days; 95% CI, −2.06 to −0.52 days) [24, 26] (Fig 2B and 2C, Table 2, and Supplements 3 and 4 in S1 File).

#### Oxygen requirements at 28 days of age and at 36 weeks PMA and incidence of moderate-to-severe bronchopulmonary dysplasia (BPD) at 36 weeks PMA

Two [15, 24], three [15, 24–25], and two [24, 26] RCTs investigated the impact of oral vitamin A supplements on oxygen requirements at 28 days of age, at 36 weeks PMA, and with moderate-to-severe BPD at 36 weeks PMA, respectively. Low-quality evidence revealed no significant differences in the oxygen required during either period or in the incidence of moderate-to-severe BPD with or without oral vitamin A supplementation (RR, 0.56; 95% CI, 0.12–2.64; RR, 0.65; 95% CI, 0.33–1.31; RR, 0.53; 95% CI, 0.07–4.17, respectively; Figs 2D, 2E and 3C, Table 2, and Supplements 3 and 4 in S1 File).

#### Postnatal steroids needed

Two RCTs [15, 26] found no significant difference in the need for steroids when oral vitamin A supplementation was administered versus the placebo, with moderate-quality evidence (RR, 1.16; 95% CI, 0.81–1.66; Fig 2F, Table 2, Supplements 3 and 4 in S1 File).

#### Hospitalization duration (days)

Three RCTs found that oral vitamin A use did not affect hospital stay lengths [24–26], with low-quality evidence (MD, −10.21 days; 95% CI, −35.99 to 15.56 days). Subgroup analysis of higher vitamin A doses showed no significant differences in these outcomes (MD, 0.93 days; 95% CI, −6.62 to 8.49 days; Fig 3B, Table 2, and Supplements 3 and 4 in S1 File).

#### Death

All four RCTs [15, 24–26] found that oral vitamin A supplements did not reduce mortality, with moderate-quality evidence (RR, 0.83; 95% CI, 0.59–1.18; Fig 3A, Table 2, Supplements 3 and 4 in S1 File).

#### Other outcomes

*Retinopathy of prematurity (ROP) requiring treatment*. Moderate-quality evidence revealed that oral vitamin A supplementation did not significantly affect the incidence of ROP requiring treatment. None of the four RCTs found a statistically significant reduction in ROP treatment requirements (RR, 0.70; 95% CI, 0.35–1.40; Fig 3D, Table 2, Supplements 3 and 4 in S1 File) [15, 24–26].

*Sepsis and late-onset sepsis*. Two [24, 26] and two [24, 25] RCTs provided data on sepsis and late-onset sepsis, respectively. Low-quality evidence showed that oral vitamin A supplementation did not reduce these outcomes (RR, 0.88; 95% CI, 0.62–1.26 and RR, 0.52; 95% CI, 0.27–1.01, respectively; Supplements 3–5 in S1 File).

*Necrotizing enterocolitis (NEC) ≥ stage 2*. Two RCTS [24, 26] showed no significant effect on the occurrence of NEC (RR, 0.70; 95% CI, 0.14–3.51) but with low certainty (Supplements 3–5 in S1 File).

*Serum retinol concentrations*. Three RCTs [24–26] monitored serum retinol levels (μg/dL) at 28 days of age. Low-quality evidence showed that oral vitamin A supplementation did not improve serum retinol levels compared with those of the placebo group (MD, 26.56 μg/dL; 95% CI, −2.36 to 55.48 μg/dL). However, subgroup analysis revealed that administration timing affected this outcome; giving as daily dose significantly affected the vitamin A levels compared with those of the placebo group (MD, 13.98 μg/dL; 95% CI, 9.81 to 18.15 μg/dL; Fig 3E, Table 2, Supplements 3 and 4 in S1 File).

#### Adverse drug-related reactions

*Vomiting*. Three RCTs with moderate-quality evidence found no statistically significant evidence that oral vitamin A increased vomiting (RR, 0.43; 95% CI, 0.13–1.39) [15, 24, 25].

*Increased intracranial pressure*. Two RCTs found that neither group experienced increased intracranial pressure regardless of whether they received oral vitamin A supplementation [24, 25].

## Discussion

Vitamin A is an essential micronutrient for preterm infants; it is crucial for proper respiratory, visual, cardiovascular, immune, and gastrointestinal functions [28] and is required for normal growth and development [4]. Extremely premature infants are born with low vitamin A stores and are at high risk of vitamin A deficiency. However, the optimal vitamin A supplementation is unknown for this population, and despite evidence of benefits, early oral vitamin A supplementation is uncommon in preterm infants [4]. Consequently, our meta-analysis mainly focused on whether oral vitamin A supplementation influences preterm outcomes.

In terms of respiratory effects, low-quality evidence with high heterogeneity (I^2^, 70%) revealed that oral vitamin A supplementation did not affect mechanical ventilation duration. Thus, we used a subgroup analysis of the higher-dosage groups, using a 2700 IU/day cutoff calculated from the weight of the smallest baby in the 3rd–5th percentile (mean body weight minus 2 standard deviations) receiving 5000 IU/kg/day of vitamin A [15]. Participants in one RCT [24] received 10,000 IU every other day; participants in another RCT received 5,000 IU every day [26]. The study’s lower-dosage group received 1500/IU of vitamin A per day [25]. The mechanical ventilation duration was not reduced regardless of oral vitamin A dosage.

Low-quality evidence revealed that oral vitamin A supplementation did not affect the duration of oxygen use (I^2^, 99%). Subgroup analysis of higher and lower dosages decreased this heterogeneity (I^2^, 0%), and the group that received a higher dosage had a slightly shorter duration of oxygen use. Oral vitamin A supplementation did not reduce the outcomes for oxygen use at 28 days or 36 weeks PMA and moderate-to-severe BPD at 36 weeks PMA, with low certainty. Moderate-quality evidence showed that oral vitamin A supplementation did not reduce the need for postnatal steroids. Respiratory results showed that oral vitamin A supplementation yielded a slightly shorter but statistically significant CPAP/HHFNC duration compared with that of the placebo group. Tyson et al. [5] assessed the efficacy of 5,000 IU IM injections three times weekly for 4 weeks and found decreases in death and BPD at 36 weeks PMA. However, that study was conducted in 1999; perinatal care has since evolved, and several modalities for improving respiratory outcomes have been established. Tolia et al. [29] found that the US nationwide shortage of injectable vitamin A did not affect the incidences of death or BPD during 2010–2012. Darlow et al. [3], Araki et al. [30], and Ding et al. [31] previously published meta-analyses that included both IM and oral vitamin A supplementation, with only one oral vitamin A supplementation study [15] in each meta-analysis. Previous meta-analyses [3, 30, 31] found that vitamin A affected BPD at 36 weeks in survivors. This discrepancy may be because our meta-analysis focused on oral vitamin A supplementation, and three of our four included studies were published between 2019 and 2021 [24–26]; in which the new BPD strategies have since been established globally. While submitting this manuscript, Rakshasbhuvankar et al. [32] published a meta-analysis on vitamin A supplementation in very preterm infants, demonstrating that oral vitamin A supplementation is as effective as other routes of administration in infants with a baseline vitamin A intake of 1500 IU/kg/day. However, because our study categorized BPD outcomes as oxygen requirement at 36 weeks PMA and moderate-severe BPD at 36 weeks PMA, we found no advantage in these outcomes.

Death was another important outcome, and none of the studies included [5–13,15,27] in the previous meta-analyses [3, 30–32] for pooled effects of death showed that vitamin A administered via any route lowered mortality. Consistent with previous study and meta-analyses [3, 29–32], oral vitamin A supplementation did not significantly reduce the incidence of death. Furthermore, our study showed that oral vitamin A supplementation did not reduce hospitalization times.

Consistent with previous meta-analyses [3, 30–32], oral vitamin A supplementation did not significantly reduce the ROP requiring treatment from four RCTs [15,24–26] with moderate certainty. In our meta-analysis, oral vitamin A supplementation did not affect sepsis, late-onset sepsis, or NEC, which is consistent with the findings of Ding et al. [31].

Because total parenteral nutrition (TPN) is routinely used and considered the standard of care for preterm infants, vitamin A is included in standard TPN. When preterm infants can be fed enterally, they can receive vitamin A through breast milk, fortified breast milk, or formula, thus providing them with a basic amount of vitamin A. Furthermore, our meta-analysis found no statistically significant difference in serum retinol levels at 28 days of age between infants receiving oral vitamin A supplementation vs. placebos (I^2^ = 99%). Subgroup analysis of the administration interval (daily or alternating days) indicated that daily administration may improve serum retinol levels (I^2^ = 0).

Whether additional oral vitamin A supplementation affects clinical outcomes is unknown. Our meta-analysis established that oral vitamin A supplementation may be beneficial only in slightly reducing the duration of noninvasive ventilation. However, moderate-to-severe BPD did not decrease, nor did oxygen requirements at 28 and 36 weeks PMA. Furthermore, oral vitamin A supplementation did not affect ROP requiring treatment, other associated complications, or mortality, nor did it appear to induce significant adverse drug-related reactions such as vomiting.

Meyer et al. [33] are currently conducting enteral vitamin A supplementation trials to illustrate this strategy and support its implementation.

### Strengths and limitations

In this meta-analysis, we evaluated how oral vitamin A supplements affect various outcomes in preterm infants. Compared with previous meta-analyses, we included four RCTs, with the most pooled estimates. Additionally, the included RCTs were conducted across different countries and ethnic groups. However, the study had some limitations. First, vitamin A concentrations in TPN and feeding may have varied by institute. Second, some outcomes had a high degree of heterogeneity; however, the authors reduced this heterogeneity as much as possible through subgroup analysis. Third, one of the included studies was conducted 2 decades ago. Additionally, the population and number of studies included in the pooled estimate were small, and the type of oral vitamin A supplement used in some clinical trials was unknown. Thus, the effect of oral vitamin A supplementation on preterm infants requires further large-scale RCTs with a standardized, uniform protocol.

## Conclusions

Oral vitamin A supplementation in preterm newborns did not affect the duration of mechanical ventilation, oxygen required at 36 weeks PMA, moderate-to-severe BPD at 36 weeks PMA, or death, or provide short-term benefits. However, oral vitamin A supplementation may have a small effect on the duration of noninvasive respiratory support without causing adverse drug-related reactions.

## Supporting information

S1 File (DOCX)

S1 Checklist (DOCX)
