# Supplementary material for: Oral vitamin A supplementation in preterm infants to improve health outcomes: A systematic review and meta-analysis
Source: PLoS One. 2022 Apr 4;17(4):e0265876. doi: 10.1371/journal.pone.0265876 (PMC8979433; doi:10.1371/journal.pone.0265876)

**Supplements**

**Supplement 1. Baseline maternal and neonatal characteristics in the included studies**

**Supplement 2. Risk-of-bias summary of the included studies using the Revised Cochrane Risk-of-Bias Tool for randomized trials**

**Supplement 3. GRADE evidence profile of the evidence outcomes**

**Supplement 4. Summarized results of the included studies categorized by outcomes**

**Supplement 5. Results of the outcomes in the systematic review and meta-analysis**

**Supplement 1. Baseline maternal and neonatal characteristics in the included studies**

| **Study** | **Vitamin A** | **Control** |
| --- | --- | --- |
| **Wardle et al., 2001** | **Oral vitamin A (n = 77 )** | **Placebo (n = 77)** |
| No. (%) with data | 77 | 77 |
| Maternal age, mean (SD), years | NR | NR |
| ANC, No. (%) | NR | NR |
| Antenatal steroids, No. (%) | 59 (77) | 63 (82) |
| Non-NSVD, No. (%) | NR | NR |
| Gestational age, mean (SD), weeks | 26 (1.5)* | 26 (1.5)* |
| Birth weight, mean (SD), g | 806 (133.3)* | 782 (218)* |
| Birth weight < 750 g, No. (%) | 29 (38) | 33 (43) |
| Male, No. (%) | 37 (48) | 30 (39) |
| Apgar score at 1 min, mean (SD) | NR | NR |
| Apgar score at 5 min, mean (SD) | NR | NR |
| Received surfactant, No. (%) | 77 (100) | 76 (99) |
| **Basu et al., 2019** | **Oral vitamin A (n = 98)** | **Placebo (n = 98)** |
| No. (%) with data | 98 | 98 |
| Maternal age, mean (SD), years | 25.4 (4.2) | 25.7 (3.9) |
| ANC ≥ 3 visits, No. (%) | 64 (65.3) | 66 (67.3) |
| Antenatal steroids, No. (%) | 50 (51) | 50 (51) |
| Non-NSVD, No. (%) | 54 (55.1) | 57 (58.2) |
| Gestational age, mean (SD), weeks | 30.9 (2.9) | 30.7 (2.7) |
| Birth weight, mean (SD), g | 1185 (194) | 1163 (181) |
| Male, No. (%) | 54 (55.1) | 56 (57.1) |
| Apgar score at 1 min, mean (SD) | 8 (1.5)* | 8 (1.5)* |
| Apgar score at 5 min, mean (SD) | 9 (0.7)* | 9 (0.9)* |
| Received surfactant, No. (%) | 41 (41.8) | 43 (43.8) |
| **Sun et al., 2020** | **Oral vitamin A (n = 132)** | **Placebo (n = 130)** |
| No. (%) with data | 132 | 130 |
| Maternal age, mean (SD), years | 29.6 (6.2) | 28.7 (6.3) |
| ANC, No. (%) | NR | NR |
| Antenatal steroids, No. (%) | 89 (67.4) | 93 (71.5) |
| Non-NSVD, No. (%) | 61 (46.2) | 59 (45.4) |
| Gestational age, mean (SD), weeks | 26.8 (1.9) | 27.1 (2.0) |
| Birth weight, mean (SD), g | 981.8 (223.6) | 983.9 (216.9) |
| Male, No. (%) | 87 (65.9) | 83 (63.8) |
| Apgar score at 1 min, mean (SD) | NR | NR |
| Apgar score at 5 min, mean (SD) | NR | NR |
| Received surfactant, No. (%) | 87 (65.9) | 89 (68.5) |
| **Rakshasbhuvankar et al., 2021** | **Oral vitamin A (n = 94)** | **Placebo (n = 94)** |
| No. (%) with data | 94 | 94 |
| Maternal age, mean (SD), years | NR | NR |
| ANC, No. (%) | NR | NR |
| Antenatal steroids, No. (%) | 79 (84) | 79 (84) |
| Non-NSVD, No. (%) | 48 (51) | 65 (69) |
| Gestational age, mean (SD), weeks | 25.8 (1.4) | 26 (1.3) |
| Birth weight, mean (SD), g | 853 (201) | 852 (211) |
| Male, No. (%) | 49 (52) | 49 (52) |
| Apgar score at 1 min, mean (SD) | NR | NR |
| Apgar score at 5 min, mean (SD) | NR | NR |
| Apgar score < 7 at 5 min , No. (%) | 29 (31) | 27 (28) |
| Received surfactant, No. (%) | NR | NR |

**Abbreviations:** ANC, antenatal care; NSVD, normal spontaneous vaginal delivery; NR, not reported; SD, standard deviation

*Converted data from median (interquartile range) to mean (SD)

**Supplement 2. Risk-of-bias summary of the included studies using Revised Cochrane Risk-of-Bias Tool for randomized trials**

| **Author (year)** | **Was the allocation sequence adequately generated?** | **Was the allocation adequately concealed?** | **Blinding: Was knowledge of the allocated interventions adequately prevented?** | | | | | **Was loss to follow-up (missing outcome data) infrequent?** | **Are reports of the study free of selective outcome reporting?** | **Was the study apparently free of other problems that could put it at a risk of bias?** |
| --- | --- | --- | --- | --- | --- | --- | --- | --- | --- | --- |
|  |  |  | Were patients blinded? | Were healthcare providers blinded? | Were data collectors blinded? | Were outcome assessors blinded? | Were data analysts blinded? |  |  |  |
| Wardle et al. (2001) | Y | Y | Y | Y | Y | Y | Y | Y | Y | PY |
| Basu et al. (2019) | Y | Y | Y | Y | PN | Y | PN | Y | PY | PY |
| Sun et al. (2020) | Y | PN | Y | Y | PY | Y | PN | Y | Y | PY |
| Rakshasbhuvankar et al. (2021) | Y | Y | Y | Y | PY | Y | PN | Y | Y | PY |
| Abbreviations: PN, probably no; PY, probably yes; N, definitely no; Y, definitely yes  Footnote: ^1^Definitely yes and probably yes were defined as low risk of bias; definitely no and probably no were defined as high risk of bias. | | | | | | | | | | |

**Supplement 3. GRADE evidence profile of the evidence outcomes**

| **Patient or population:** Preterm infants  **Intervention:** Oral vitamin A  **Comparison:** Placebo, no treatment, or usual care | | | | | | | | | | | | | | | | | | |
| --- | --- | --- | --- | --- | --- | --- | --- | --- | --- | --- | --- | --- | --- | --- | --- | --- | --- | --- |
| **Study design** | **No. of studies** | **Certainty assessment** | | | | | | | | | **No. of participants** | | | | **Effect** | | | |
|  |  | **Risk of bias** | **Inconsistency** | | | **Indirectness** | **Imprecision** | | **Other considerations** | | **Oral vitamin A supplements** | | **Placebo** | | **Estimation of absolute effects** | | | **Certainty** |
|  |  |  |  |  |  |  |  |  |  |  |  |  |  |  | **Risk**  **(95% CI)** | **Absolute (95% CI)** | |  |
| **Efficacy of oral vitamin A supplementation** | | | | | | | | | | | | | | | | | | |
| **Duration of mechanical ventilation (days)** | | | | | | | | | | | | | | | | | | |
| RCT | 4 | Not serious | | Serious | Not serious | | | Serious | | None | | 401 | 399 | - | | | MD **1.07 lower** (2.98 lower to 0.83 higher) | ⨁⨁⊝⊝ LOW |
| **Duration of CPAP or HHFNC (days)** | | | | | | | | | | | | | | | | | | |
| RCT | 2 | Not serious | | Not serious | Not serious | | | Not serious | | None | | 192 | 192 | - | | | MD **0.96 lower** (1.59 lower to 0.33 lower) | ⨁⨁⨁⨁ HIGH |
| **Duration of oxygen requirement (days)** | | | | | | | | | | | | | | | | | | |
| RCT | 3 | Not serious | | Serious | Not serious | | | Serious | | None | | 324 | 322 | - | | | MD **7.4 lower** (23.13 lower to 8.33 higher) | ⨁⨁⊝⊝ LOW |
| **Moderate-to-severe BPD at 36 weeks PMA** | | | | | | | | | | | | | | | | | | |
| RCT | 2 | Not serious | | Serious | Not serious | | | Serious | | None | | 36/192 (18.8%) | 39/192 (20.3%) | **RR 0.53** (0.07–4.17) | | | **95 fewer per 1,000** (from 189 fewer to 644 more) | ⨁⨁⊝⊝ LOW |
| **Oxygen requirement at 36 weeks PMA** | | | | | | | | | | | | | | | | | | |
| RCT | 3 | Not serious | | Serious | Not serious | | | Serious | | None | | 67/307 (21.8%) | 90/305 (29.5%) | **RR 0.65** (0.33–1.31) | | | **103 fewer per 1,000** (from 198 fewer to 91 more) | ⨁⨁⊝⊝ LOW |
| **Postnatal steroids needed** | | | | | | | | | | | | | | | | | | |
| RCT | 2 | Not serious | | Not serious | Not serious | | | Serious | | None | | 44/171 (25.7%) | 38/171 (22.2%) | **RR 1.16** (0.81–1.66) | | | **36 more per 1,000** (from 42 fewer to 147 more) | ⨁⨁⨁ MODERATE |
| **Death** | | | | | | | | | | | | | | | | | | |
| RCT | 4 | Not serious | | Not serious | Not serious | | | Serious | | None | | 43/401 (10.7%) | 52/399 (13.0%) | **RR 0.83** (0.59–1.18) | | | **22 fewer per 1,000** (from 53 fewer to 23 more) | ⨁⨁⨁ MODERATE |
| **Duration of hospitalization (days)** | | | | | | | | | | | | | | | | | | |
| RCT | 3 | Not serious | | Serious | Not serious | | | Serious | | None | | 324 | 322 | - | | | MD **10.21 lower** (35.99 lower to 15.56 higher) | ⨁⨁⊝⊝  LOW |
| **Oxygen requirement at 28 days of age** | | | | | | | | | | | | | | | | | | |
| RCT | 2 | Not serious | | Serious | Not serious | | | Serious | | None | | 45/175 (25.7%) | 51/175 (29.1%) | **RR 0.56** (0.12–2.64) | | | **128 fewer per 1,000** (from 256 fewer to 478 more) | ⨁⨁⊝⊝  LOW |
| **ROP requiring treatment** | | | | | | | | | | | | | | | | | | |
| RCT | 4 | Not serious | | Not serious | Not serious | | | Serious | | None | | 15/401 (3.7%) | 23/399 (5.8%) | **RR 0.70** (0.35–1.40) | | | **17 fewer per 1,000** (from 37 fewer to 23 more) | ⨁⨁⨁ MODERATE |
| **Sepsis** | | | | | | | | | | | | | | | | | | |
| RCT | 2 | Not serious | | Not serious | Serious | | | Serious | | None | | 51/192 (26.6%) | 58/192 (30.2%) | **RR 0.88** (0.62–1.26) | | | **36 fewer per 1,000** (from 115 fewer to 79 more) | ⨁⨁⊝⊝ LOW |
| **Late onset sepsis** | | | | | | | | | | | | | | | | | | |
| RCT | 2 | Not serious | | Not serious | Serious | | | Serious | | None | | 12/230 (5.2%) | 23/228 (10.1%) | **RR 0.52** (0.27–1.01) | | | **48 fewer per 1,000** (from 74 fewer to 1 more) | ⨁⨁⊝⊝ LOW |
| **NEC ≥ stage 2** | | | | | | | | | | | | | | | | | | |
| RCT | 2 | Not serious | | Not serious | Serious | | | Serious | | None | | 5/192 (2.6%) | 7/192 (3.6%) | **RR 0.70** (0.14–3.51) | | | **11 fewer per 1,000** (from 31 fewer to 92 more) | ⨁⨁⊝⊝ LOW |
| **Level of serum retinol concentration** | | | | | | | | | | | | | | | | | | |
| **Serum retinol concentration at 28 days old (µg/dL)** | | | | | | | | | | | | | | | | | | |
| RCT | 3 | Not serious | | Serious | Not serious | | | Serious | | None | | 239 | 225 | - | | | MD **26.56 higher** (2.36 lower to 55.48 higher) | ⨁⨁⊝⊝ LOW |
| **Adverse drug-related reaction** | | | | | | | | | | | | | | | | | | |
| **Vomiting** | | | | | | | | | | | | | | | | | | |
| RCT | 3 | Not serious | | Not Serious | Not serious | | | Serious | | None | | 4/307 (1.3%) | 10/305 (3.3%) | **RR 0.43** (0.13–1.39) | | | **19 fewer per 1,000** (from 29 fewer to 13 more) | ⨁⨁⨁ MODERATE |
| **Abbreviations:** BPD, bronchopulmonary dysplasia; CI, confidence interval; CPAP, continuous positive airway pressure; HHNC, humidified high-flow nasal cannula; MD, mean difference; NEC, necrotizing enterocolitis; PMA, postmenstrual age; RCT, randomized controlled trial; ROP, retinopathy of prematurity; RR, risk ratio | | | | | | | | | | | | | | | | | | |

**Supplement 4. Summarized results of the included studies categorized by outcomes**

| **Authors (year)** | **Intervention** | **Oral vitamin A** | **Control** | | **Mean difference (95% CI)** |
| --- | --- | --- | --- | --- | --- |
|  |  | **Mean ± SD** | **Mean ± SD** | |  |
| **Efficacy of oral vitamin A supplementation** | | | | | |
| **Duration of mechanical ventilation (days)** | | | | | |
| ***Higher dosage (≥2,700 IU/day****)* | | | | | |
| Wardle et al., 2001 | Oral vitamin A | 14 ± 14.81* | 15 ± 19.26* | | \| −1.00 [−6.43, 4.43] \| \| --- \| |
| Basu et al., 2019 | Oral vitamin A | 4.5 ± 2.77* | 4.3 ± 6.11* | | 0.20 [−1.13, 1.53] |
| Rakshasbhuvankar et al., 2021 | Oral vitamin A | 2.75 ± 19.29* | 3.33 ± 12.84* | | −0.58 [−5.26, 4.10] |
| Subtotal (95% CI)  Heterogeneity: Tau² = 0.00; χ² = 0.26, df = 2 (P = 0.88); I² = 0%  Test for overall effect: Z = 0.13 (P = 0.90) | | | | | 0.08 [−1.16, 1.33] |
| ***Lower dosage (<2,700 IU/day****)* | | | | | |
| Sun et al., 2020 | Oral vitamin A | 4.3 ± 3.7 | 6.7 ± 4.2 | | −2.40 [−3.36, −1.44] |
| Subtotal (95% CI)  Heterogeneity: Not applicable  Test for overall effect: Z = 4.91 (P < 0.00001) | | | | | -2.40 [−3.36, −1.44] |
| Total (95% CI)  Heterogeneity: Tau² = 2.06; χ² = 9.85, df = 3 (P = 0.02); I² = 70%  Test for overall effect: Z = 1.11 (P = 0.27) | | | | | −1.07 [−2.98, 0.83] |
| **Duration of CPAP or HHFNC (days)** | | | | | |
| Basu et al., 2019 | Oral vitamin A | 2.5 ± 1.26* | 3.5 ± 2.96* | | −1.00 [−1.64, −0.36] |
| Rakshasbhuvankar et al., 2021 | Oral vitamin A | 59.67 ± 16.02* | 59.17 ± 11.6* | | 0.50 [−3.50, 4.50] |
| Heterogeneity: Tau² = 0.00; χ² = 0.53, df = 1 (P = 0.47); I² = 0%  Test for overall effect: Z = 3.00 (P = 0.003) | | | | | −0.96 [−1.59, −0.33] |
| **Duration of oxygen requirement (days)** | | | | | |
| ***Higher dosage (≥2,700 IU/day)*** | | | | | |
| Basu et al., 2019 | Oral vitamin A | 4.35 ± 2.05* | 5.65 ± 3.31* | | −1.30 [−2.07, −0.53] |
| Rakshasbhuvankar et al., 2021 | Oral vitamin A | 49.79 ± 58.46* | 48.17 ± 47.56* | | 1.62 [−13.61, 16.85] |
| Subtotal (95% CI)   \| Heterogeneity: Tau² = 0.00; χ² = 0.14, df = 1 (P = 0.71); I² = 0%  Test for overall effect: Z = 3.29 (P = 0.0010) \| \| --- \| | | | | | −1.29 [−2.06, −0.52] |
| ***Lower dosage (<2,700 IU/day****)* | | | | | |
| Sun et al., 2020 | Oral vitamin A | 23.1 ± 5.4 | 43.4 ± 10.6 | | −20.30 [−22.34, −18.26] |
| Subtotal (95% CI)  Heterogeneity: Not applicable  Test for overall effect: Z = 19.49 (P < 0.00001) | | | | | −20.30 [−22.34, −18.26] |
| Total (95% CI)  Heterogeneity: Tau² = 176.33; χ² = 291.62, df = 2 (P < 0.00001); I² = 99%  Test for overall effect: Z = 0.92 (P = 0.36) | | | | | −7.40 [−23.13, 8.33] |
| **Duration of hospitalization (days)** | | | | | |
| ***Higher dosage (≥2,700 IU/day)*** | | | | | |
| Basu et al., 2019 | Oral vitamin A | 12 ± 4.44* | 14 ± 9.63* | | −2.00 [−4.10, 0.10] |
| Rakshasbhuvankar et al., 2021 | Oral vitamin A | 108 ± 29.63* | 102 ± 28.89* | | 6.00 [−2.37, 14.37] |
| Subtotal (95% CI)  Heterogeneity: Tau² = 22.32; χ² = 3.30, df = 1 (P = 0.07); I² = 70%  Test for overall effect: Z = 0.24 (P = 0.81) | | | | | 0.93 [−6.62, 8.49] |
| ***Lower dosage (<2,700 IU/day****)* | | | | | |
| Sun et al., 2020 | Oral vitamin A | 30.1 ± 6.3 | 64.2 ± 7.5 | | −34.10 [−35.78, −32.42] |
| Subtotal (95% CI)  Heterogeneity: Not applicable  Test for overall effect: Z = 39.82 (P < 0.00001) | | | | | −34.10 [−35.78, −32.42] |
| Total (95% CI)  Heterogeneity: Tau² = 512.31; χ² = 588.61, df = 2 (P < 0.00001); I² = 100%  Test for overall effect: Z = 0.78 (P = 0.44) | | | | | −10.21 [−35.99, 15.56] |
| **Serum retinol concentration at 28 days of age (µg/dL)** | | | | | |
| ***Administer daily*** | | | | | |
| Sun et al., 2020 | Oral vitamin A | 35 ± 24.92 | 20.16 ± 13.44 | | 14.84 [10.00, 19.68] |
| Rakshasbhuvankar et al., 2021 | Oral vitamin A | 26.4 ± 11.26* | 14.9 ± 12.96* | | 11.50 [3.29, 19.71] |
| Subtotal (95% CI)  Heterogeneity: Tau² = 0.00; χ² = 0.47, df = 1 (P = 0.49); I² = 0%  Test for overall effect: Z = 6.57 (P < 0.00001) | | | | | \| 13.98 [9.81, 18.15] \| \| --- \| |
| ***Administer every other day*** | | | | | |
| Basu et al., 2019 | Oral vitamin A | 74.9 ± 16.9 | 21.9 ± 7.9 | | 53.00 [49.05, 56.95] |
| Subtotal (95% CI)  Heterogeneity: Not applicable  Test for overall effect: Z = 26.29 (P < 0.00001) | | | | | 53.00 [49.05, 56.95] |
| Total (95% CI)  Heterogeneity: Tau² = 644.04; χ² = 177.83, df = 2 (P < 0.00001); I² = 99%  Test for overall effect: Z = 1.80 (P = 0.07) | | | | | 26.56 [-2.36, 55.48] |
| **Author** | **Intervention** | **Oral vitamin A** | **Control** | | **Risk Ratio  (95% CI)** |
|  |  | **Event/Total** | **Event/Total** | |  |
| **Oxygen requirement at 28 days of age** | | | | | |
| Wardle et al., 2001 | Oral vitamin A | 43/77 | 42/77 | | 1.02 [0.77, 1.36] |
| Basu et al., 2019 | Oral vitamin A | 2/98 | 9/98 | | 0.22 [0.05, 1.00] |
| Heterogeneity: Tau² = 1.00; χ² = 4.27, df = 1 (P = 0.04); I² = 77%  Test for overall effect: Z = 0.73 (P = 0.47) | | | | | 0.56 [0.12, 2.64] |
| **Oxygen requirement at 36 weeks PMA** | | | | | |
| ***Study period ≥ 10 years ago*** | | | | | |
| Wardle et al., 2001 | Oral vitamin A | 40/77 | 37/77 | | 1.08 [0.79, 1.48] |
| Heterogeneity: Not applicable  Test for overall effect: Z = 0.48 (P = 0.63) | | | | | 1.08 [0.79, 1.48] |
| ***Study period <10 years ago*** | | | | | |
| Basu et al., 2019 | Oral vitamin A | 2/98 | 9/98 | | 0.22 [0.05, 1.00] |
| Sun et al., 2020 | Oral vitamin A | 25/132 | 44/130 | | 0.56 [0.37, 0.86] |
| Heterogeneity: Tau² = 0.12; χ² = 1.37, df = 1 (P = 0.24); I² = 27%  Test for overall effect: Z = 2.06 (P = 0.04) | | | | | 0.47 [0.23, 0.96] |
| Total (95% CI)  Heterogeneity: Tau² = 0.26; χ² = 9.62, df = 2 (P = 0.008); I² = 79%  Test for overall effect: Z = 1.21 (P = 0.23) | | | | | 0.65 [0.33, 1.31] |
| **Moderate-to-severe BPD at 36 weeks PMA** | | | | | |
| Basu et al., 2019 | Oral vitamin A | 0/98 | 4/98 | | 0.11 [0.01, 2.04] |
| Rakshasbhuvankar et al., 2021 | Oral vitamin A | 36/94 | 35/94 | | 1.03 [0.71, 1.48] |
| Heterogeneity: Tau² = 1.53; χ² = 2.37, df = 1 (P = 0.12); I² = 58%  Test for overall effect: Z = 0.60 (P = 0.55) | | | | | 0.53 [0.07, 4.17] |
| **Postnatal steroids needed** | | | | | |
| Wardle et al., 2001 | Oral vitamin A | 30/77 | 26/77 | | 1.15 [0.76, 1.76] |
| Rakshasbhuvankar et al., 2021 | Oral vitamin A | 14/94 | 12/94 | | 1.17 [0.57, 2.39] |
| Heterogeneity: Tau² = 0.00; χ² = 0.00, df = 1 (P = 0.98); I² = 0%  Test for overall effect: Z = 0.79 (P = 0.43) | | | | | 1.16 [0.81, 1.66] |
| **Death** | | | | | |
| ***Higher dosage (≥2,700 IU/day)*** | | | | | |
| Wardle et al., 2001 | Oral vitamin A | 25/77 | 29/77 | | 0.86 [0.56, 1.33] |
| Basu et al., 2019 | Oral vitamin A | 9/98 | 16/98 | | 0.56 [0.26, 1.21] |
| Rakshasbhuvankar et al., 2021 | Oral vitamin A | 8/94 | 5/94 | | 1.60 [0.54, 4.71] |
| Subtotal (95% CI)  Heterogeneity: Tau² = 0.03; χ² = 2.43, df = 2 (P = 0.30); I² = 18%  Test for overall effect: Z = 0.79 (P = 0.43) | | | | \| 0.84 [0.55, 1.29] \| \| --- \| | |
| ***Lower dosage (<2,700 IU/day****)* | | | | | |
| Sun et al., 2020 | Oral vitamin A | 1/132 | 2/130 | | 0.49 [0.05, 5.36] |
| Subtotal (95% CI)  Heterogeneity: Not applicable  Test for overall effect: Z = 0.58 (P = 0.56) | | | | 0.49 [0.05, 5.36] | |
| Total (95% CI)  Heterogeneity: Tau² = 0.00; χ² = 2.62, df = 3 (P = 0.45); I² = 0%  Test for overall effect: Z = 1.03 (P = 0.30) | | | | | 0.83 [0.59, 1.18] |
| **Sepsis** | | | | | |
| Basu et al., 2019 | Oral vitamin A | 20/98 | 28/98 | | 0.71 [0.43, 1.18] |
| Rakshasbhuvankar et al., 2021 | Oral vitamin A | 31/94 | 30/94 | | 1.03 [0.68, 1.56] |
| Heterogeneity: Tau² = 0.01; χ² = 1.25, df = 1 (P = 0.26); I² = 20%  Test for overall effect: Z = 0.68 (P = 0.50) | | | | | 0.88 [0.62, 1.26] |
| **Late-onset sepsis** | | | | | |
| Basu et al., 2019 | Oral vitamin A | 9/98 | 18/98 | | 0.50 [0.24, 1.06] |
| Sun et al., 2020 | Oral vitamin A | 3/132 | 5/130 | | 0.59 [0.14, 2.42] |
| Heterogeneity: Tau² = 0.00; χ² = 0.04, df = 1 (P = 0.84); I² = 0%  Test for overall effect: Z = 1.94 (P = 0.05) | | | | | 0.52 [0.27, 1.01] |
| **ROP requiring treatment** | | | | | |
| ***Higher dosage (≥2,700 IU/day)*** | | | | | |
| Wardle et al., 2001 | Oral vitamin A | 6/77 | 6/77 | | 1.00 [0.34, 2.96] |
| Basu et al., 2019 | Oral vitamin A | 1/98 | 2/98 | | 0.50 [0.05, 5.42] |
| Rakshasbhuvankar et al., 2021 | Oral vitamin A | 6/94 | 6/94 | | 1.00 [0.33, 2.99] |
| Subtotal (95% CI)  Heterogeneity: Tau² = 0.00; χ² = 0.29, df = 2 (P = 0.86); I² = 0%  Test for overall effect: Z = 0.18 (P = 0.86) | | | | | \| 0.94 [0.45, 1.95] \| \| --- \| |
| ***Lower dosage (<2,700 IU/day****)* | | | | | |
| Sun et al., 2020 | Oral vitamin A | 2/132 | 9/130 | | 0.22 [0.05, 0.99] |
| Subtotal (95% CI)  Heterogeneity: Not applicable  Test for overall effect: Z = 1.97 (P = 0.05) | | | | | 0.22 [0.05, 0.99] |
| Total (95% CI)  Heterogeneity: Tau² = 0.04; χ² = 3.23, df = 3 (P = 0.36); I² = 7%  Test for overall effect: Z = 1.01 (P = 0.31) | | | | | \| 0.70 [0.35, 1.40] \| \| --- \| |
| **NEC ≥ stage 2** | | | | | |
| Basu et al., 2019 | Oral vitamin A | 1/98 | 4/98 | | 0.25 [0.03, 2.20] |
| Rakshasbhuvankar et al., 2021 | Oral vitamin A | 4/94 | 3/94 | | 1.33 [0.31, 5.80] |
| Heterogeneity: Tau² = 0.53; χ² = 1.59, df = 1 (P = 0.21); I² = 37%  Test for overall effect: Z = 0.43 (P = 0.67) | | | | | 0.70 [0.14, 3.51] |
| **Adverse drug-related reactions** | | | | | |
| **Vomiting** | | | | | |
| Wardle et al., 2001 | Oral vitamin A | 1/77 | 5/77 | | 0.20 [0.02, 1.67] |
| Basu et al., 2019 | Oral vitamin A | 3/98 | 5/98 | | 0.60 [0.15, 2.44] |
| Sun et al., 2020 | Oral vitamin A | 0/132 | 0/130 | | Not estimable |
| Heterogeneity: Tau² = 0.00; χ² = 0.73, df = 1 (P = 0.39); I² = 0%  Test for overall effect: Z = 1.41 (P = 0.16) | | | | | 0.43 [0.13, 1.39] |
| **Increased intracranial pressure** | | | | | |
| Basu et al., 2019 | Oral vitamin A | 0/98 | 0/98 | | Not estimable |
| Sun et al., 2020 | Oral vitamin A | 0/132 | 0/130 | | Not estimable |
| Heterogeneity: Not applicable  Test for overall effect: Not applicable | | | | Not applicable | |

**Abbreviations:** BPD, bronchopulmonary dysplasia; CI, confidence interval; CPAP, continuous positive airway pressure; HHFNC, humidified high-flow nasal cannula; NEC, necrotizing enterocolitis; PMA, postmenstrual age; ROP, retinopathy of prematurity; SD, standard deviation

*Converted data from median (interquartile range) to mean (SD)

**Supplement 5. Results of the outcomes in the systematic review and meta-analysis**

**A. Sepsis**


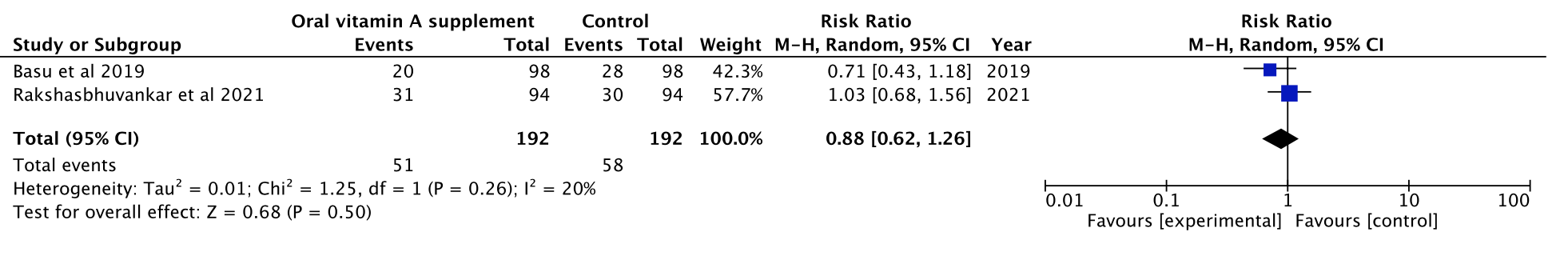


**B. Late-onset sepsis**


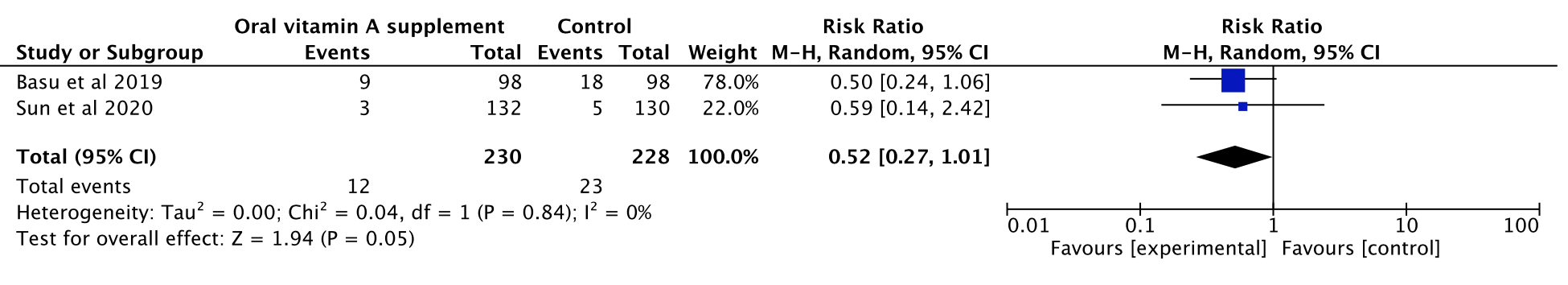


**C. NEC ≥ stage 2**


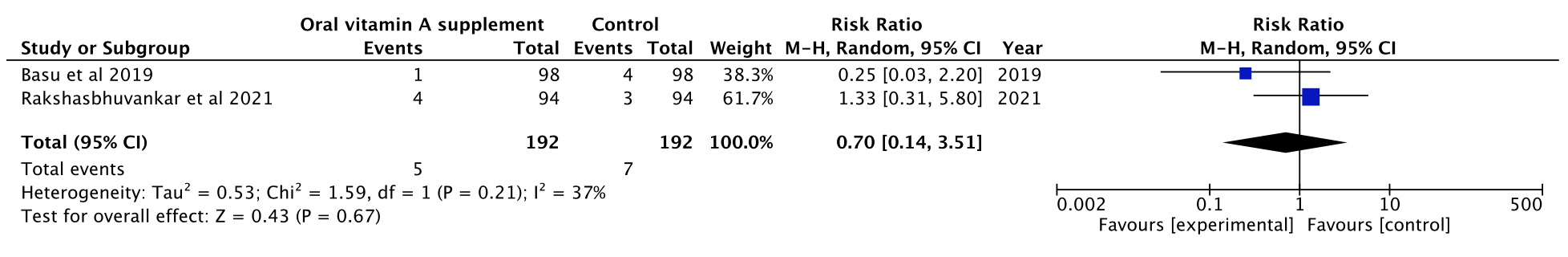

Supplement: S1 File — (DOCX) [file pone.0265876.s001.docx]
